# Chicago Medical Society

**Published:** 1886-03

**Authors:** 


### Society Reports.

### CHICAGO MEDICAL SOCIETY.

Officially Reported for the ATLANTA MEDICAL AND SURGICAL JOURNAL.

STATED MEETING, January 18, 1886.

President *pro tem.*, D. W. Graham, M. D., in the chair.

Dr. E. J. Doering read a paper entitled

### MUTUAL PROTECTION AGAINST BLACKMAIL.

The author stated that among the many trials which physicians have to encounter in the practice of their profession is the ever-existing liability of being blackmailed. This may either assume the more frequent form of a so-called malpractice suit, or the relatively less frequent charge of a criminal assault, according to the viciousness of the complainant. Such suits against physicians are increasing. One reason quoted was the fact that every city is overrun with petty lawyers, who have little or nothing to do, and are always willing to encourage any suit whatever, if there be the least prospect of getting something out of the defendant. The author stated that since investigating the matter he became convinced that many of these blackmail schemes were settled before being made public. Many a physician preferred being robbed of one or two hundred dollars rather than incur the publicity, the loss of time and the endless expense of a lawsuit. Again, the average jury, composed of the ignorant and illiterate, will always have a strong leaning toward the complainant and against the defendant in a malpractice suit, as physicians are popularly supposed to be capitalists. The author stated that personally he had never been sued or even threatened with a suit, and it was therefore from no motive of selfish interest, but from sincere regard for the welfare of the profession, that he advocated the formation of an association for the mutual protection of physicians against blackmailing suits of all kinds. His plan is to organize a society composed of two or three hundred members of the regular profession, all of whom shall be of acknowledged ability, possessing a good moral character and standing in the community—said association to employ the best legal talent obtainable, by the year, to furnish the members such legal advice as they may desire at any time and defend any suit against the members arising in the discharge of their professional duties. It was stated that the expense to each member of an association composed of about two hundred would not exceed five dollars per annum, and that an initiation fee of five dollars would create a sufficient fund for court expenses. Such an association would be a power in preventing suits. Let it be known that the individual physician is backed by the financial and moral support of a few hundred of the best physicians, and aided by the best legal talent obtainable, and he will be let severely alone by the offscouring and dregs of society who constitute, almost without exception, the blackmailing element in our professional life. The author stated that he was not aware of the existence of such an association as the one proposed in any other city, but the principle at least had been carried out recently by the New York County Medical Society in voting $500 to assist in the defense of the Drs. Purdy, members of the Society, in the case of Brown *vs.* Purdy. After reading a number of letters from prominent physicians in favor of forming a protective association, and presenting several legal opinions sustaining the advisability, practicability and legal status of such a society, the author concluded by stating his firm belief that such an association for mutual protection was needed—that it would be a power for good—that it would draw the profession closer together—that, in short, it would be based on the principles of a common brotherhood, viz., equality, harmony, justice and unity.

Dr. F. C. Hotz said that the extract of his letter to Dr. Doering, which was incorporated in the paper, indicated that at the time it was written he did not think favorably of the project; and, after listening with much interest to the doctor's arguments, he saw no reason for changing his opinion. Professional reputation and honor is the most personal of all personal property; if he lost it, it does not hurt anybody but himself, and therefore if any attack be made on it he should certainly wish to employ among the able lawyers the one in whose ability he had the greatest confidence. But he was not sure whether the lawyer retained by this protective union would be the one to whom he should like to trust the defense of his reputation. The attorney might be as able, or abler, than the lawyer of his own choice; but should the case go against him, he should never feel satisfied that the lawyer had done all that could be done for him unless he had full confidence in him. It is with the lawyer, as with the physician, a question of confidence, and his patrons find no fault with his treatment as long as they have implicit faith in his ability.

An objection of greater weight, however, has been urged by several of the doctor's correspondents in asking what possible effect it might have if the fact was brought out in court that the defendant belonged to such a union? The lawyers whose opinions were obtained and read by the doctor say it cannot legally affect the case. There is no doubt but that this is true. But the verdict of a jury in malpractice suits is not determined by the legal aspect of the case; and circumstances which cannot have any legal effect upon the case have often made a deep impression upon a jury and decided the case against the physician.

Very interesting was that part of the paper in which the doctor evolved his idea how this new society could prevent, ward off, malpractice suits. He believes the shysters would not be so eager to engage in this business if they knew they had to fight a corporation with plenty of means to employ the best legal talent. Why this should discourage those fellows it is hard to understand. They do not sue poverty-stricken doctors. Whom they select for their victims they suppose to be rich and consequently able to employ a good lawyer. They do not expect to have an easy game; but why should they not try it? They don't risk anything by it. The blackmailer's stake is only two dollars and a half for filing his application, and his lawyer's stake is his time, which is not worth much anyhow. So you see they have nothing to lose, but much to gain. What difference should it make to them whether the opposing counsel is engaged by one physician or by one hundred? If you wish to devise means by which this blackmailing nuisance can be stopped, or at least reduced to a minimum, you must try to get to the roots of the evil; that is, you must find the causes which usually bring it forth; and you will not go far to find them; for you find them right at your door, in our own profession, in the shape of *indiscriminate dispensation of gratuitous services and of unkind remarks of one physician about another.* Physicians are altogether too quick to give their services gratis to almost anybody at any time. But you know very well people do not value very much what they can get for the mere asking; they do not think much of what they get for nothing. And it is also a widespread notion (especially among the lower, uneducated people) that the quality of service is regulated by the amount of money they pay for it; that the treatment at a free dispensary, because gratuitous, is not the same, not as good as at the physician's office where they have to pay for it. These people cannot persuade themselves that a physician will take the same interest in a case whether or not he is paid for his services. The poor, therefore, are always suspicious that they do not get their full share of attention; they are quickly ready to charge their physician with carelessness if the case goes wrong. And with a patient in this frame of mind it takes but very little encouragement to begin a suit for damages. And in nine out of ten cases, doubtless, this encouragement is furnished by the members of our own profession. He did not mean to charge physicians with purposely, willfully instigating a lawsuit against a professional brother. Though this has been done, such extraordinary baseness is a rare exception.

Stop running each other down; stand by each other; sustain each other; "stick together and be clanish;" let it be understood in public that no reputable physician will prostitute himself by going to court as expert for a blackmailer. If all the reputable physicians of this city adopt and act on this principle, blackmailing the medical profession would soon be a thing of the past, and malpractice suits more effectually prevented than by the organization of a protective union.

Dr. P. S. Hayes said that from his costly experience in a malpractice suit, he felt that an association such as suggested by Dr. Doering would be of great service. The lawyer employed by such an association would speedily acquire such a fund of medical knowledge that he would be considered an expert in malpractice cases. He would not require an amount of coaching necessary to prepare for any given case as would be requisite in the case of a lawyer who had had no experience in such cases. His opportunity for obtaining information in a given case would be largely extended, for each member of the association to whom he might apply would be interested in giving him the desired knowledge. He would soon become acquainted with medical witnesses and know which would give the best testimony in any case.

An association of the character suggested by the paper might be a means of educating its members in regard to laws bearing on the rights of physicians and their patients now not generally understood.

For one he is heartily in favor of such an association, and should give it his hearty support.

Dr. G. C. Paoli said Dr. Doering's paper is not only a valuable one, but contains such a high, noble, charitable feeling that the Society ought to be grateful to him. He wondered that such steps had not been taken before, because so many of our professional brethren have not only suffered annoyance, but pecuniary loss as well. How can we expect from an ignorant jury a decision based on scientific knowledge and justice?

Dr. F. M. Weller said that the subject of the paper was worthy of consideration; that the discussion of the formation of an association with an object so widely different from the Medical Society seemed out of place—the one essentially scientific, the other in the nature of an insurance. The right to form such an organization was unquestioned; the policy should be considered by each individual; that while any one might be made the object of blackmail, he believed that charges of malpractice more frequently arose from the ignorance of physicians of the statutes affecting the practice of medicine, especially those of the criminal code, and of the rulings of the courts in cases.

